# Supplementary material for: Thoracic Tumor Associated with a Unilateral Empyema in a Beef Cow: A Case Report
Source: Vet Sci. 2023 May 27;10(6):376. doi: 10.3390/vetsci10060376 (PMC10302296; doi:10.3390/vetsci10060376)
Supplement: Supplementary file 1 [file vetsci-10-00376-s001.zip › vetsci-2402246-supplementary.pdf]

Supplemental Table S1. Values for biochemical analysis.

Parameter	Value	Reference range
Fibrinogen (g/L)	3.9	[2 – 7]
Total proteins (g/L)	84	[67 – 75]
Albumin (g/L)	25	[28 – 34]
Urea (mmol/L)	6.2	[2.1 – 7.9]
Creatinine (µmol/L)	91	[44 – 97]
ASAT (U/L)	97	[28 – 100]
CK (U/L)	127	[56 – 1,236]
GGT (U/L)	58	[22 – 64]
Bilirubin (µmol/L)	3.7	[2 – 10]

ASAT = aspartate aminotransferase; CK = creatine kinase; GGT = gamma-glutamyl transferase;U/L = units per litter.

Supplemental Table S2. Values for haematological analysis.

Parameter	Value	Reference range	
		Absolute	Relative
Haematocrit (%)	26.8	[24 – 39]	
Haemoglobin (g/L)	89	[82 – 130]	
RBC (.10^{12}/L)	6.57	[4.8 – 7.8]	
WBC (.10^{9}/L)	9.15	[4.4 – 10.8]	
Neutrophil granulocytes (.10^{9}/L)	5.95 (65 %)	[0.8 – 5]	[20 % – 40 %]
Eosinophil granulocytes (.10^{9}/L)	0.46 (5 %)	[0.1 – 2.1]	[1 % – 15 %]
Basophil granulocytes (.10^{9}/L)	0 (0 %)	[0 – 0.1]	0 %
Lymphocytes (.10^{9}/L)	2.56 (28 %)	[1.8 – 4.9]	[40 % – 60 %]
Monocytes (.10^{9}/L)	0.18 (2 %)	[0.3 – 1.2]	[2 % – 10 %]
Platelets (.10^{3}/L)	951	[120 – 600]	

RBC = red blood cells   WBC = white blood cells.

Supplemental Table S3. Values for the cytological analysis of the intra-thoracic liquid collection.

Parameter	Value
Cellularity (/µL)	8,820
Neutrophil granulocytes (%)	89
Macrophages (%)	7
Eosinophil granulocytes (%)	0
Lymphocytes (%)	4
Proteins (g/L)	55

Supplemental Table S4. Primary antibodies utilized for immunohistochemistry.

Antibodies	Antigenic retrieval	Dilution	Incubation	Reference Company
Vimentin V9	30′ 96°C Low pH	1/50	50′ at room temperature	M0725, Dako
Desmin D33	30′ 96°C High pH	1/50	50′ at room temperature	M0760, Dako
Actin HHF35	None	1/25	50′ at room temperature	M0635, Dako
Cytokeratin MNF116	30′ 96°C Low pH	1/300	50′ at room temperature	M0821, Dako
